# What matters to patients? A mixed method study of the importance and consideration of oncology patient demands

**DOI:** 10.1186/s12913-021-06247-0

**Published:** 2021-03-20

**Authors:** Mathias WAELLI, Etienne Minvielle, Maria Ximena Acero, Khouloud Ba, Benoit Lalloué

**Affiliations:** 1 EA MOS 7348, EHESP, French School of Public Health, 15 Avenue du Professeur Léon Bernard, 35043 Rennes, France; 2 Global Health Institute, University of Geneva, 24 Rue du Général-Dufour, 1211 Genève 4, Switzerland; 3 i3-CRG Ecole Polytechnique, Route de Saclay, 91120 Palaiseau, France; 4 Gustave Roussy, 114 Rue Edouard Vaillant, 94800 Villejuif, France; 5 Assistance Publique-Hôpitaux de Paris, 3 Avenue Victoria, 75004 Paris, France; 6 BIGS Université de Lorraine CNRS INRIA IECL F54000, 615 Rue du Jardin-Botanique, 54600 Villers-lès-Nancy, France

**Keywords:** Patient demands, Organisation, Oncology, Patient-centred care, Care customisation

## Abstract

**Background:**

A patient-centred approach is increasingly the mandate for healthcare delivery, especially with the growing emergence of chronic conditions. A relevant but often overlooked obstacle to delivering person-centred care is the identification and consideration of all demands based on individual experience, not only disease-based requirements. Mindful of this approach, there is a need to explore how patient demands are expressed and considered in healthcare delivery systems.

This study aims to: (i) understand how different types of demands expressed by patients are taken into account in the current delivery systems operated by Health Care Organisations (HCOs); (ii) explore the often overlooked content of specific non-clinical demands (i.e. demands related to interactions between disease treatments and everyday life).

**Method:**

We adopted a mixed method in two cancer centres, representing exemplary cases of organisational transformation: (i) circulation of a questionnaire to assess the importance that breast cancer patients attach to every clinical (C) and non-clinical (NC) demand identified in an exploratory inquiry, and the extent to which each demand has been taken into account based on individual experiences; (ii) a qualitative analysis based on semi-structured interviews exploring the content of specific NC demands.

**Results:**

Further to the way in which the questionnaires were answered (573 answers/680 questionnaires printed) and the semi-structured interviews (36) with cancer patients, results show that NC demands are deemed by patients to be almost as important as C demands (C = 6.53/7 VS. NC = 6.13), but are perceived to be considered to a lesser extent in terms of pathway management (NC = 4.02 VS C = 5.65), with a significant variation depending on the type of non-clinical demands expressed. Five types of NC demands can be identified: demands relating to daily life, alternative medicine, structure of the treatment pathway, administrative and logistic assistance and demands relating to new technologies.

**Conclusions:**

This study shows that HCOs should be able to consider non-clinical demands in addition to those referring to clinical needs. These demands require revision of the healthcare professionals’ mandate and transition from a supply-orientated system towards a demand-driven approach throughout the care pathway. Other sectors have developed hospitality management, mass customisation and personalisation to scale up approaches that could serve as inspiring examples.

## Highlights

- HCOs must increasingly address non-clinical, lifestyle-related patient demands along the entire care pathway.
- These non-clinical demands are considered important by oncology patients but are not adequately taken into account by HCOs.
- There are 5 types of non-clinical demands that could be better addressed for oncology patients.
- The incorporation of these demands into the healthcare structure could be inspired by examples from other service sectors.

## Background

With an increasing number of patients affected by chronic conditions, healthcare organisations (HCOs), from primary care clinics to integrated health systems, are compelled to redefine the process and scope of their delivery system and be more patient-centred. This approach means giving organisational responses to all demands expressed by patients along the entire care pathway, including hospitalisation, ambulatory care, transportation to and from nursing home or home) [1]. Nonetheless, the incorporation of all patient demands into the healthcare delivery system poses a challenge. Healthcare delivery systems are mainly designed around diseases and other clinical conditions [2–4], and healthcare professionals generally translate patient demands into clinical needs based on their medical knowledge (one such example is personalized medicine where the clinical needs of patients, particularly in terms of therapy, are defined by integrating the molecular and genetic characteristics of the patient) [5–7].

However, “non-clinical demands” exist. For instance, the social determinants of a patient (social isolation or financial barriers to care access) can lead to demands being addressed during management of the care pathway (e.g. transportation, home meal delivery services). The integration of these psychosocial needs is a current issue for healthcare delivery systems, in particular for vulnerable patients whose social situation impacts their clinical condition. This can lead to the necessity of identifying high need cost patients [8]. In addition, patients also express demands during the care process based on their preferences - i.e. ideas, expectations, values -, that Patient Centred Care (PCC) experts [9–11] have often claimed to be ignored in care delivery systems.

Patients express demands related to aspects of their lives that interact with the management of the disease. This can be helped through administrative procedures or questions regarding adaptation of transportation needs, and a whole set of services generally poorly apprehended during care, and which can be linked to the field of hospitality management [12]. A lack of global consideration for these various types of demands can result in a fragmented healthcare system and an inefficient use of meagre resources [3, 13]. In order to initiate the design and implementation of a demand-driven organisational model, it is necessary to consider and study the content of all non-clinical demands, and their association with clinical demands, an area that has hitherto been investigated to a lesser extent [14, 15].

The purpose of this article is twofold:

(i) It initially aims to assess the importance of each demand expressed by patients and the extent to which these demands are taken into account in managing their care pathway. Two categories of demands are defined in support of the analysis: clinical (C), and non-clinical (NC) demands and it is assumed that, as a general rule, greater consideration is given to clinical demands as opposed to non-clinical demands.
(ii) The article then seeks to investigate the content of specific non-clinical demands in greater depth.

We use the concept of “demands” rather than “needs” or “unmet needs”, two concepts that are often difficult to define, depending on the context in which they are applied and the disciplines involved [16]. The notion of “demands” departs from these concepts in two ways. Firstly, we concentrate on demands perceived as opposed to expressed, normative and comparative concepts of need [17]. Secondly, demands point to a lack of well-being which might (but need not) indicate an unmet need for care. It follows that the concept of demand does not involve an express need for care. Demands are only a starting point for looking at what patient personally perceive as difficulties and complaints [18].

This research focuses on two Health Care Organisations (HCOs) specialising in oncology. Cancer is a significant example of chronic care, illustrating the need to consider various patient demands. Indeed, cancer patients who are increasingly exposed to chronic conditions are involved in care pathways, which include numerous return trips between home and the health care establishment. They also experience stressful changes in their general health throughout the care continuum. This involves regular updates in the management of their care pathways and ongoing consideration of the interaction between clinical and non-clinical demands.

The two selected HCOs have developed innovative patient paths based on remote patient monitoring systems, including nurse navigators (NNs). The principal role of NNs is to co-ordinate patient management activities. As pathway ambassadors, NNs are in direct contact with patients before, during and after periods of hospitalisation. They must, therefore, pay close attention to all expressed and non-expressed patient demands along the entire care pathway. In so doing, they provide interesting areas of exploration in understanding which demands patients can express along their care pathway and how these are taken into account in the HCO healthcare delivery system.

This study was driven by an inductive clinical reasoning model to better understand how clinical and non-clinical patient demands are integrated in an organizational process. The first step served to understand the importance of all demands (clinical and non-clinical), and then see how these demands were incorporated into current modern health care delivery systems. The results obtained in the first step prompted us to explore the content of non-clinical demands in a second step, which was a deep empirical identification and description of non-clinical demands. Through these 2 steps, we sought to illustrate the usefulness of this analysis for delivering care and services at patient pathway level, while providing additional insight into the consideration of non-clinical demands in the patient-provider relationship during the clinical decision-making process [19, 20].

## Methods

### Setting

We developed our study in specific HCOs, namely two cancer centres. HCO 1 is a major cancer centre. In the year of the study (2017), HCO 1 had 414 beds, 14,600 admissions, 6133 consultations and 3314 patients were included in the navigation programme. HCO 2 is a cancer centre including a teaching hospital in western France. In the year of the study, HCO 2 had 103 beds, 7612 admissions and 2867 consultations.

### Research design

We adopted a mixed method comprising two phases. Based on exploratory research involving HCO1 and HCO2, we designed a quantitative approach (Phase 1) using a questionnaire to address the first objective (how patient demands are taken into account by healthcare organisations). With regard to the second objective, namely the content of non-clinical demands, we adopted a qualitative analysis (semi-structured interviews) (Phase 2). We adopted a “demand driven” perspective as opposed to “supply driven” one. We started with the evaluation of the patient demands before defining the organizational response, during both phases. In phase 1, HCO 1 is the main field and HCO 2 the field for confirmatory analysis, whereas in phase 2, HCO 1 is the unique field of exploration.

#### Phase 1. Quantitative phase: analysis of the importance and consideration of patient demands

In phase 1, we assessed the importance of each demand and the extent to which patients perceived that their demand had been taken into account.

### Questionnaire design

Based on exploratory interviews and observations of interaction of oncology NNs with patients in both HCOs, and our experience as field experts, we collectively identified 27 general patient demands classified into two types, clinical and non-clinical. For each demand (except the one relating to “anticipated directive” that could not be evaluated), we formulated one question to evaluate the perceived importance and one question to evaluate how it has been taken into account in the HCO. Consequently, the questionnaire comprised 52 items that assess the importance and consideration of each demand based on an 7-item Likert scale (from 1 for “not important at all” or “not taken into account at all” to 7 for “extremely important” or “fully taken into account”) regarding patient characteristics (age, household, income, professional status, SPC (social professional category), education, follow-up period, satisfaction with lifestyle), and ultimately, an open-ended question for potential additional comments (a full version of the questionnaire is appended). The questionnaire was pretested on two patients.

### Patient sample

In order to boost the significance of the outcome of the questionnaire, which investigates importance and consideration regardless of condition, we decided to focus this study phase on a specific population: breast cancer patients in a chronic situation. We applied the following inclusion criteria: (i) women aged 18 and over; residing in metropolitan France; (ii) life expectancy of over 6 months confirmed by health professionals, already monitored in the HCO; (iii) participant in one of the innovative follow-up programmes. The following exclusion criteria were applied: (i) non- French speaking patients; (ii) patients who have not yet received cancer treatment (just diagnosed with cancer).

### Administration of the questionnaire

On the main site (HCO 1), the self-administered questionnaire was given to patients attending their consultation from 09/05/2017 to 23/05/2017, by nurses from the outpatient consultation service of the oncology department. A ballot box was provided for patients to submit their completed anonymous questionnaires.

On the second site (HCO 2), the questionnaires were given to day hospital, radiology and consultation personnel to be self-administered by patients under the same baseline conditions from 01/06/2017 to 30/09/2017.

### Statistical analysis

A descriptive analysis was carried out for each HCO. Number and percentage of the different modalities were used to describe the characteristics of the patients in each HCO (with the exception of age, which was studied using the average). Fisher’s exact tests were used to compare the categorical characteristics between the two HCO, and Wilcoxon tests to compare the age. Wilcoxon test was preferred to a t-test because we wanted to avoid any issues caused by non-normality when designing the study protocol, as the sample size for HCO2 was moderately small. Answers relating to importance and consideration were processed as quantitative scores (from 0 to 7 points), and studied using the average value across the respondents of each HCO separately. Analyses were carried out with R 3.4.3 software.

#### Phase 2. Qualitative phase: specification of non-clinical demands

##### Sampling

In order to specify, in depth, the type of various non-clinical demands, which were identified and evaluated in the previous phase, we conducted semi-structured interviews with patients presenting all types of cancer in order to understand the common content of these demands under different conditions. We carried out this study in HCO 1, applying the same inclusion and exclusion criteria as in Phase 1, from January to March 2018 (KB) and from June to July 2019 (MW).

##### Data collection

Potential participants were recruited on site by KB and then by MW in the hall of HCO 1. After introducing the study, an appointment was made, mostly for the same day. Semi structured interviews were conducted in a dedicated room in HCO 1 (duration: 1 h on average). They were based on an interview grid listing the previous phase 1 non-clinical demands, with a focus on challenging aspects for the management of care pathways Discussions remained very open. At times it took a very narrative turn, as the patient started to describe their pathways from the beginning to the day of the interview. The interviewer also asked for precisions and examples concerning the different needs encountered at each step of the process.

##### Data analysis

The 36 interviews were recorded, transcribed in full and analysed gradually as data collection took place. Data analysis followed an inductive process. This included data familiarisation, open coding and definition of categories of demands. To increase validity, three researchers performed the analysis separately, then discussed their results to reach a consensus. First, KB, MW and EM individually read each interview to have a sense of the whole. Then, they identified meaning units composed of specific demands. Each meaning unit was then assigned a code (see related needs. Table 1). The codes were discussed during monthly meetings, which allowed to create a code list of 18 related needs, and classify them in five categories of non-clinical demands expressed by patients. Data collection ceased when no new codes or categories could be identified.

**Table 1:** 5 Categories of Non-Clinical Demands

Categories of demands	Related needs (No. of occurrences/36)	Feed-back/r
**Category 1: Demands relating to daily lifestyle during the treatment period**	• Need for home assistance (5/36)	*Need for* ***home assistance from time to time****, yes I do it, I was so tired, especially as I live alone, that I can’t do anything at home. You become very delicate due to side effects (68 years-old female with breast cancer, PE teacher*).
	• Need for physical exercise (15/36)	I usually do a lot of sport. It’s important to strike a balance. I generally run a lot. Up to last year, I was taking part in half-marathons. Now, it’s complicated. I need exercise that is more suitable for my condition (67 years-old male with prostate cancer).
	• Need for pet-sitting (3/36)	In terms of activities, I used to do a lot of sport before. Nowadays, with the cancer and heavy treatment, I’m very frustrated because I can’t do sport any more … I need to do it**!** … (32 years-old female with breast cancer, no profession)
	• Need for hotel services (3/36)	The first time I was admitted to hospital it was an emergency. We didn’t have time to see it coming. My husband couldn’t stay long with me in the emergency unit because we have a small dog. And he stayed at home. In the days that followed, we had to reach a solution with neighbours so that he could come to see me without leaving the dog on its own for too long. These are all little things, but they add up. I’m the one who usually manages this type of thing at home and I know my husband found it difficult to suddenly have to cope with all that. (72 years-old female with colorectal cancer).
	• Need for entertainment (6/36)	My friend lives more than 3 h away by road. When she first came here with me, she had to find a place to stay. And the hotels around here are a bit grim. Fortunately, last time, she was able to stay with a friend in Paris. That’s better of course but it’s a bit worrying when she has to travel back alone from here in the evening (61 years-old male with throat cancer).
	• Need for transport information (2/36)	When you spend a lot of time here, you sometimes want to leave your room for a change of scenery. And stroll through the corridors – not exactly ideal! The nurses and the doctors are very nice. The staff generally do all they can to really make us comfortable. But I would like to have access to a library in the building. I love books. (56 years-old male with lung cancer). A bus operates between here (HCO 1) and the station, but it stops on the other side of the car park. And if you want to see the timetable, it’s only displayed over there. It would be nice for the buses to stop at the entrance to the building so that we don’t have to stand and wait outside (58 years-old female with breast cancer).
**Category 2: Demands relating to alternative medicine requirements and improved well-being excluding prescriptions issued by healthcare professionals**	• Need for pain management not prescribed by healthcare professionals during consultations within the scope of therapeutic protocols: acupuncture, auriculotherapy, magnetic therapy, etc. (15/36)	I reached a point where I couldn’t take any more medication to counteract the side effects of the treatments. I needed to have a more natural form of treatment – acupuncture and hypnosis. I think that’s helping me a lot today (63 years-old female patient with breast cancer).
**Category 3: Demands relating to the organisational aspect of the treatment pathway**	• Need for appointments in line with patient constraints. (17/36)	My daughter had to travel with her job. So it made sense for me to stay in the south to look after my grand-daughter until the end of the week. I called the co-ordination nurse to find out if I could rearrange my appointment. Fortunately, this was possible … (68 years-old female with breast cancer).
	• Need for direction (27/36)	When I got the news, my life was turned upside down. I felt lost. The hospital is like a giant maze. I didn’t understand anything. I didn’t want to go from one appointment to the next like a ping pong ball. I’ve stuck to that a bit. I needed to be given the chance to be independent. I called the co-ordination nurse - not for her to arrange my appointments but to get the contact details of my doctor’s secretary (53 years-old female with uterine cancer).
	• Need for a contact person (16/36)	When you call HCO1, there’s no-one to take care of you. No contact! No follow-up. I was alone! I would like to have **someone to contact when I can** to ask them questions and help me … (64 year-old female with breast cancer, PE teacher).
	• Need for improved co-ordination between treatment stakeholders (13/36)	I have my chemotherapy at home. I need a perfusion pump for that. On the one hand, you’re waiting for the “chemo ok” and, on the other hand, for the nurse who has to administer it (…) The problem is that the two are not always co-ordinated. Sometimes the nurse comes in the morning but the equipment isn’t there. The nurse therefore comes back later. The outcome – I spend all day waiting (44 years-old female with breast cancer).
**Category 4: Demands for administrative and logistic assistance**	• Need for social assistance (19/36)	My situation is **very difficult** to manage alone. My **husband is unemployed** … .. I need to see someone from **social care** and work out with them how to manage our **financial situation**. (32 years-old female with breast cancer, no profession).
	• Need for administrative and legal assistance in socially complex cases (11/36)	When you have the disease, your income stops. You have to expend an incredible amount of energy to manage all the red tape (sickness insurance, provident/pension fund, health funds, hospital, etc.). And patients are too weak to manage all that on their own. They need to be guided. They need to be told, there you are, do it like that! (64 years-old male, plasmacytoma, IT engineer)
	• Need for information regarding reimbursement methods for treatment-related costs (8/36)	You’re fighting on all fronts. I’ve just had a colectomy. I could hardly move. I couldn’t see myself doing any housework. I requested home assistance. The nurse referred me to social assistance who told me that I wasn’t covered because of my income. Finally, a friend advised me to contact my complementary health insurance company directly. I called them and they told me that they would cover 4 h of cleaning each week. But I didn’t know that before (70 years-old female with colorectal cancer).
	• Need for prostheses or equipment and medical equipment (9/36)	I didn’t give much thought to a hair prosthesis at first. It was my son’s reaction that convinced me. He had seen a TV programme about cancer where the women were bald. That made a big impression on him. He was afraid that his friends would see his mother like that. So I was given the contact details of a specialist providing this type of wig. There isn’t a great deal of choice – it’s mostly for older ladies. I bought one, but it wasn’t me and I didn’t really like it. I used to wear it when I went out, especially for my son, but at home I wore a scarf. Thankfully it didn’t last long (44 years-old female with breast cancer)
**Category 5: Demands relating to the use of new information and communication technologies (NTIC) and to telemedicine**	• Use of the Internet to find information (30/36)	I learned a lot on the Internet. There were lots of accounts from people who have experienced exactly the same thing and who pass on information. And it’s not the same as nurses or doctors. That helped me a lot. It got me thinking a bit, even about saving money. For instance, hair prostheses, wigs, are very expensive and there’s virtually no cover. So on the forums, women offer to resell ones they have bought. That convinced me not to buy one (59 years-old woman with lung cancer).
	• Use of the Internet to interact with health professionals (9/36)	My doctor always answers emails. That’s very important. I am more comfortable with this than the phone. I get the impression that it doesn’t bother him as much either. (56 years-old male with colorectal cancer).
	• Telemedicine (23/36)	It’s good to know that I can contact someone if I have any concerns. As I’m alone, I really don’t know what to do sometimes. But I don’t want this (follow-up phone call from the co-ordinating nurse) all the time (68 year-old female with breast cancer, PE teacher).

## Results

### Phase 1. Analysis of importance/consideration

In HCO 1, the survey covering 26 demands was made available to 613 patients. In total, 114 patients refused to answer or did not comply with the inclusion criteria (patients undergoing screening, non-French speakers, etc.). Overall, 499 questionnaires were completed and submitted.

In HCO 2, 74 questionnaires out of 130 distributed to health professionals were completed and handed in: 44 for radiology, 16 for the day hospital and 14 for consultations.

#### Characteristics of respondents

Patients from both sites were similar in terms of average age (approximately 55 years of age, *p* = 0.42) and size of household (number of adults *p* = 0.40, number of dependent children *p* = 0.12). Most of them were couples (*p* = 0.13) without CMU (Universal Health Insurance Cover)(*p* = 1.00). There were, however, significant differences between the two sites (as HCO 1 patients were generally monitored for longer, *p* < 0.001), the level of qualification (HCO 1 patients were slightly more qualified, *p* = 0.01), professional status (a large proportion of HCO 2 patients were on sick leave, *p* = 0.04) and SPC (social-professional category) (more executives and intellectual professions in HCO 1, *p* < 0.001) (see Appendix 2).

#### Comparison between importance and consideration perceived

**Fig. 1:**
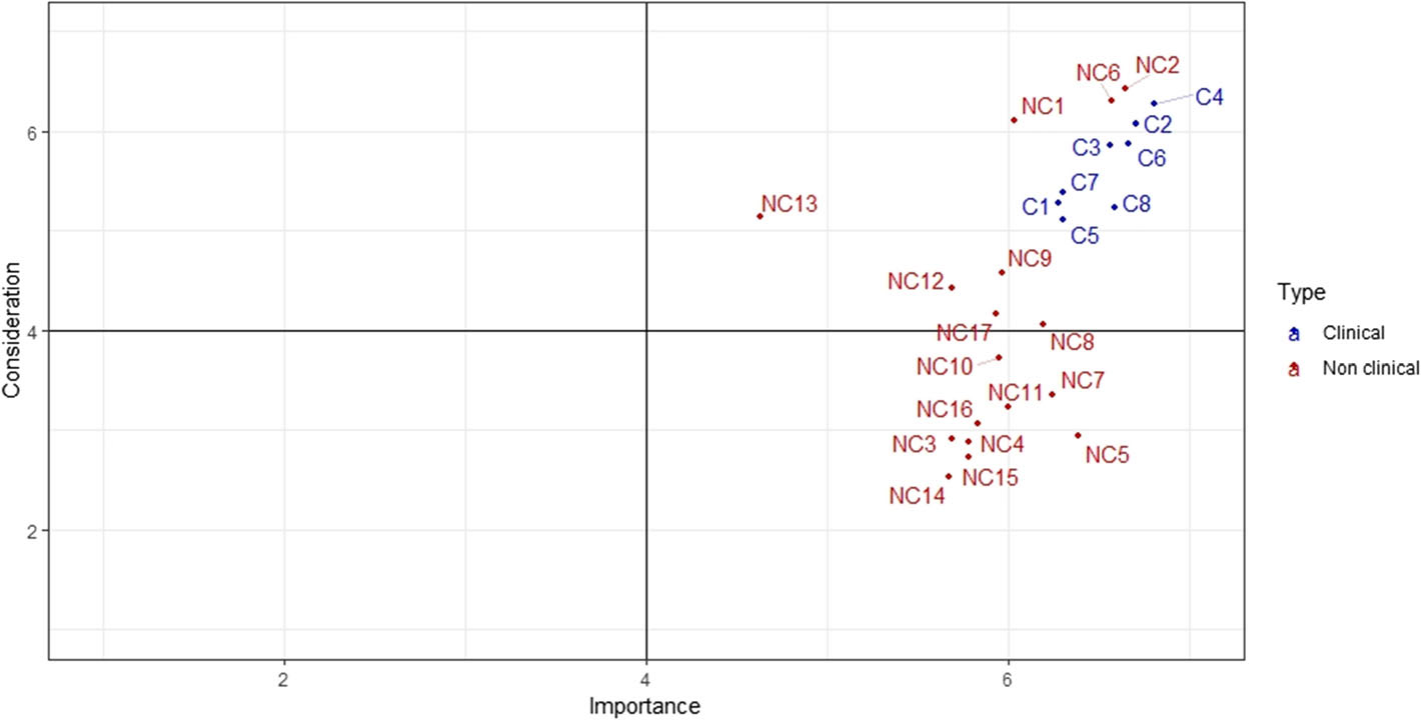
HCO 1. Results of the importance and consideration given to demands. CF. List of the 26 demands, Appendix 1

**Fig. 2:**
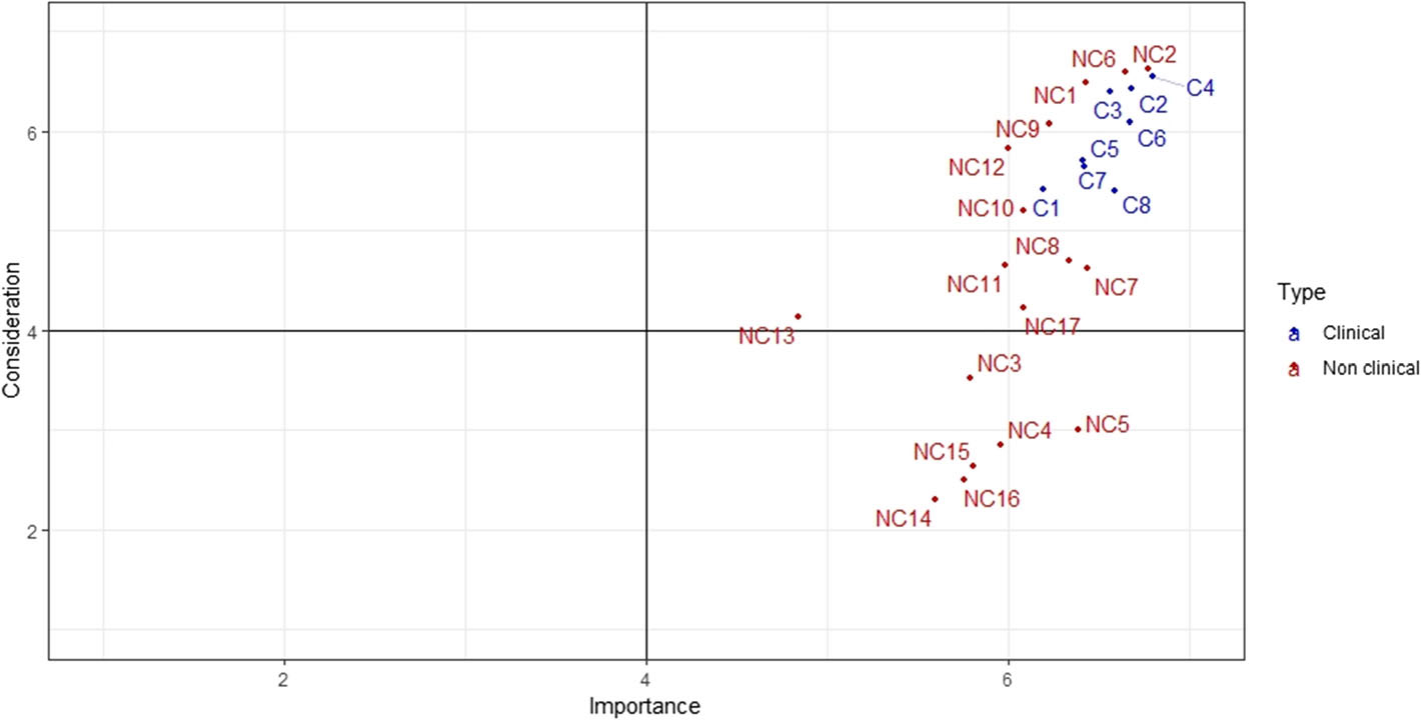
HCO 2. Results of the importance and consideration given to demands. Cf. List of the 26 demands Appendix 1

**Fig. 3:**
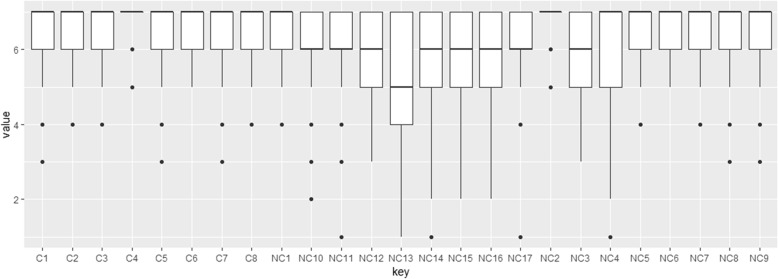
Variations in consideration of demands perceived by patients. Cf. List of the 26 demands Appendix 1

As a general rule, considerable importance is given to all demands in both HCOs (average: 6.14). Six types of demands nevertheless still appear to be more important than others: adaptation of treatment in line with patient requirements (6.8), pain management (6.7), professional courtesy (6.7), patient information in terms of treatment choice and changes (6.7), respect for intimacy (6.6), the adaptation of paramedical care (6.6) and access to cutting-edge treatments (6.6). These response profiles are similar for both sites (Figs. 1 and 2). One demand is considered less important on both sites: consideration of the patient’s beliefs (4.7). However, it is also the demand that varies the most in terms of importance, depending on the patients concerned (see Fig. 3).

In contrast, patients’ opinions regarding the way in which these demands were taken into consideration during their treatment varies to greater extent. Demands which scored highly in this respect are: staff courtesy (6.5), the adaptation of treatment in line with patient requirements (6.3), respect for intimacy (6.3), the fact that professionals provide contact details (6.2) and pain management (6.1). HCO 2 patients mostly found that the adaptation of paramedical care (6.4) and timing adjustments in line with patient requirements (6.1) were given appropriate consideration.

According to patients, less consideration was given to the following requirements: knowledge of tariff options (fees, procedures, accommodation) during hospital stays (3.0), access to legal assistance (3.0), access to administrative assistance (2.9) or social support (2.9), advance knowledge of levels of reimbursement (2.7) and advance availability of tariffs for therapeutic procedures (2.5). A certain variability in responses is noted, which is greater for non-clinical demands particularly when they involve very personal questions relating to care values (e.g. NC13, see Fig. 3).

### Results phase 2. In-depth understanding of the content of the non-clinical demands

The patients interviewed during the third phase were mostly women (21/36) between 32 and 72 years of age. The most common type of cancer was gynaecological cancer (14/36) with most patients presenting breast cancer (12/14). A large number of patients received oral chemotherapy (24/36).

These interviews allowed us to classify non-clinical demands according to 5 categories determined by the nature of the demand in response to a specific requirement (see Table 1): 1. Demands related to lifestyle during the treatment period, 2. Demands related to alternative medicine requirements and improved well-being, excluding prescriptions issued by healthcare professionals, 3. Demands related to the organisational aspect of the treatment pathway 4. Demands for administrative and logistic assistance, 5. Demands related to the use of new information and communication technologies (NTIC) and telemedicine. NC demands selected for the questionnaire were included in n°1, n°3, n°4. n°2 and n°5 emerged from phase 2.

## Discussion

Our study highlights two main points.

Firstly, it shows that non-clinical demands expressed by patients actually exist and in some cases are given less consideration than clinical demands in healthcare delivery systems. This finding is consistent with a large body of literature on patient experiences and PCC studying the quality of care from a patient perspective since 1985 [21, 22]. These studies show the importance of developing tools that can assess the patient experience rather than his/her satisfaction, and highlight the importance of considering needs of non-clinical nature (e.g. information about waiting and process time) [23]. Within this first point about non-clinical demands, the current study brings the current study providers two additional observations. It proposes first and foremost an empirical view of these non-clinical demands expressed by patients. Some of these demands may seem very similar to clinical demands, such as the use of alternative medicines, but the observation reports nuances as they are self-managed by patients, without professional involvement. Others represent aspects of everyday life with a chronic disease, a scenario frequently experienced by patients, and a far cry from patient care and clinical treatments issues. Moreover, it highlights the need to consider demands along the entire patient pathway. However, some of these NC demands are largely beyond a patient-physician relationship, such as concerns for transportation or pet-sitting. Other NC demands emphasise the need to manage this physician-patient relationship through new and remote follow-up. In view of these various perspectives, the content of these NC demands gives new insight into the traditional concept of biopsychosocial needs. The latter, defined as the motivation for achieving a satisfactory level of functioning as a human being [24], mainly focuses on care aspects and less on issues relating to everyday life in a disease scenario. More specifically, these demands also consolidate the Patient-Centred care approach by starting from the patient experience of health care delivery schedules [25].

Our results also suggest a variation in importance and experience depending on the patients and their respective demands. This applies to some NC demands in particular (NC3–4–12-17, Fig. 3). Our study stresses the need for customised answers according to the combination of C and NC demands expressed by each patient [2, 13].

Consequently, the second point emphasised by our research concern the need to consider all clinical and non-clinical demands via a customised approach. Our findings therefore questions therefore the role of HCOs in delivering more patient-centred and demand-driven delivery systems. Furthermore, the content of these demands shows that they must be considered along the entire patient pathway, and not only in the provider-patient relationship.

This resonates with well-known approaches in other service sectors, such as hospitality management, which define appropriate practices during the customer relationship in order to respond to their demands [26–28], and mass customisation [29, 30] or personalisation to scale [31, 32] proposing specific answers for each customer at affordable cost. However, important challenges have to be overcome if such approaches are to be applied to healthcare. With specific regard to the transfer of hospitality management techniques, the professional mandate must be extended beyond clinical practices. Although the structured response to customers’ demands of all types is clearly aligned in the healthcare sector [33–35], these pioneering experiences remain isolated, calling for a review of the healthcare professionals’ mandate. Some non-clinical demands are not taken into account appropriately. Logically, the distinction between those demands would seem to correspond to different healthcare priorities. However, our results highlight nuances in terms of this assumption. Some of the non-clinical demands such as “courtesy”, “intimacy” and “identification” appear to be taken into account in HCOs. These NC demands are not connected to clinical demands, but they imply the direct relationship with caregivers who generally believed these practices to be aligned with their clinical “mandate” [36, 37]. The boundaries for considering such practices within the healthcare spectrum have already been studied [38, 39], and resonate with the conventional debate between care and cure [40]. Modern activities highlight the need to develop practices more in line with these patient demands.

Development of a customised care model also requires consideration of segmentation analysis methods to incorporate clinical and non-clinical demands from a common as opposed to a primarily disease-based perspective [2, 41]. But less explicitly, it also requires answers to specific organisational questions: from fabrication (i.e. ensuring that production meets each demand), to assembly (i.e. “modular” service structures capable of combining in a flexible manner the multiple products and services required by the combination of demands), and distribution (i.e. co-ordination and integration of various assembly structures to ensure the timely delivery of a given service to the patient) [29, 42–44]. The fabrication of non-clinical demands can generate new services such as concierge services in HCOs [33–45]. Although some attempts have been made to incorporate specific “fabrication” steps or to combine services in a “modular package” [46], there is no integrative system as far as we are aware. The transition from a supply-oriented delivery system to a demand-driven approach would require further investigations in this direction.

This study has some limitations. First, in order to produce questionnaires that seemed, according to our experience in HCO1, not too long, we had to merge a certain number of situations into one main criterion (e.g. intimacy). This has led to a certain number of differences being overlooked. These differences were highlighted in conjunction with phase 2 patient interviews. Second, the study took place in two hospitals in France. This may not seem enough to assess the taking into consideration of patient demands by healthcare organization. In fact, the perception patients have on how diverse demands (including demands concerning information about organizational characteristics) are taken into consideration depends more on healthcare teams in the numerous wards concerned than on the central administration of HCOs. Third, in the first step, Non-clinical demands have been studied in in a specific case, namely breast cancer. The focus made the questionnaire more significant. However, some non-clinical demands can be disease-specific, limiting then the scope of the results. Although it represents an exemplary case in which the patient potentially expresses numerous requirements, it is worthy of being investigated in other contexts. That is why we included patients suffering from all types of cancer in the second step of the study. Last, it may seem counter-intuitive to specify the content of the demands (qualitative phase) after the questionnaire. In our case, this is linked to the research strategy adopted, namely to address, first, the consideration of different demands. The observation that numerous NC demands are not taken into account has led to a more in-depth study of their content. Our approach highlights the need to fluctuate between qualitative and quantitative research from a patient perspective.

## Conclusions

To our knowledge, this is the first empirical study that has examined the consideration of various patient demands in healthcare delivery systems. Our typology of five non-clinical demands is also an initial step to providing a better understanding of various patient preferences and demands beyond the clinical sphere. Given our focus on oncology, we encourage researchers to examine this issue in relation to other chronic conditions such as diabetes or cardiovascular diseases. The nascent and heterogeneous state of knowledge in this area is also challenging in order to make summative findings at this point based on this single research project. However, our study highlights the fact that invisible demands should be identified more clearly and integrated in a segmentation analysis of patient needs and individualised responses in order to develop new demand-driven and patient-centred approaches.

## Data Availability

The data sets used and analysed in this study are available from the corresponding author on reasonable request.

## Appendix 1

**Table 2:** List of the 26 demands identified

**Clinical**	Straightforward access to medical records	C1
	Individualised pain management for each patient	C2
	Paramedical care adapted to the requirements of each patient	C3
	Medication adapted to the requirements of each patient	C4
	Availability of additional care (psychological, dietary follow-up, physiotherapy and sophrology, etc.)	C5
	Patient information on choice/change of treatment	C6
	Consideration of the patient’s opinion in selecting/changing treatment	C7
	Access to cutting-edge treatments (targeted therapy, surgical robots, clinical trials, etc.)	C8
**Non-clinical**	Professionals make themselves available to patients	NC1
	Professionals are courteous with patients	NC2
	Access to legal assistance (in the case of disputes with the institution or a healthcare professional)	NC3
	Access to administrative assistance (reporting of condition and triggering of reimbursements)	NC4
	Access to social support	NC5
	Respecting patient intimacy	NC6
	Formalisation of anticipated directives, as necessary	/
	Application of the right to be forgotten *In France, the AERAS agreement, which provides wider access to insurance and loans to individuals with serious risks; insured parties no longer have to declare their cancer after a certain period of time following the cessation of treatment*	NC7
	Provision of a contact person for remote interaction with patients	NC8
	Adaptation of appointment schedules in line with patient constraints	NC9
	Announcement/justification of waiting times	NC10
	Shorter waiting times (consultations, wait for diagnosis, treatments)	NC11
	Availability of all information on potential activities and services (sport, beauty treatments, writing workshops, etc.)	NC12
	Consideration of patients’ beliefs *Belief refers to values and primarily religious beliefs*	NC13
	Availability in advance of details of therapeutic procedure tariffs	NC14
	Information in advance on levels of reimbursement for therapeutic procedures	NC15
	Availability of all tariff options (fees, surgical procedure, accommodation, etc.) during hospitalisation	NC16
	Availability of all tariff options for transport payments	NC17

## Appendix 2

**Table 3:** Characteristics of the respondents to the questionnaire survey

	HCO 1 (***n*** = 499)	HCO 2 (***n*** = 74)	***p***-value
*Average (SD) age*	55.2 years (SD = 13.3)	54.3 years (SD = 11.8)	0.42
*In a couple*			
Yes	342 (70.7%)	59 (79.7%)	0.13
No	142 (29.3%)	15 (20.3%)	
*With CMU (Universal Health Insurance Cover)*			
Yes	15 (3.5%)	2 (3.2%)	1.00
No	416 (96.5%)	61 (96.8%)	
*Length of follow-up*			
< 1 month	16 (3.4%)	0 (0%)	< 0.001
1–3 month(s)	33 (7.0%)	22 (30.6%)	
3–6 months	43 (9.2%)	18 (25.0%)	
6 months-1 year	59 (12.6%)	22 (30.6%)	
> 1 year	319 (67.9%)	10 (13.9%)	
*Education*			
No qualification	26 (5.9%)	4 (5.8%)	0.01
Professional diploma – Study certificate	55 (12.5%)	5 (7.2%)	
CAP-BEP	65 (14.8%)	19 (27.5%)	
Bac (French equivalent to UK “A” level)	79 (17.9%)	20 (29.0)	
Bac + 2	129 (29.3%)	13 (18.8%)	
Bac + 5 or higher	86 (19.6%)	8 (11.6%)	
*Professional status*			
In active employment	169 (36.3%)	16 (22.9%)	0.04
Retired	156 (33.5%)	21 (30.0%)	
Unemployed	18 (3.9%)	3 (4.3%)	
Student	2 (0.4%)	0 (0%)	
Sick leave/disability	99 (21.3%)	27 (38.6%)	
Out of work and not seeking employment	21 (4.5%)	3 (4.3%)	
*Socio-professional category*			
Farmer	1 (0.2%)	2 (2.9%)	< 0.001
Craftsperson, trader, company director	21 (4.7%)	2 (2.9%)	
Executives and intellectual professions	143 (32.1%)	10 (14.3%)	
Intermediate profession	66 (14.8%)	8 (11.4%)	
Employee	191 (42.8%)	42 (60.0%)	
Labourer	10 (2.2%)	6 (8.6%)	
Not affected	14 (3.1%)	0 (0%)	
*Monthly household income*			
< 1100	16 (3.5%)	3 (4.3%)	0.74
1100–1400	38 (8.4%)	5 (7.1%)	
1400–2100	73 (16.1%)	8 (11.4%)	
2100–3000	80 (17.7%)	18 (25.7%)	
3000–4100	92 (203.4%)	12 (17.1%)	
4100–5100	45 (10.0)	7 (10.0%)	
> 5100	39 (8.6%)	4 (5.7%)	
No response	69 (15.3%)	13 (18.6%)	

## Abbreviations

HCO: Healthcare organisation
C: Clinical
NC: Non-clinical
PCC: Patient centered care
